# Synergism between cAMP and PPAR**γ** Signalling in the Initiation of UCP1 Gene Expression in HIB1B Brown Adipocytes

**DOI:** 10.1155/2013/476049

**Published:** 2013-03-11

**Authors:** H. Y. Chen, Q. Liu, A. M. Salter, M. A. Lomax

**Affiliations:** School of Biosciences, Division of Nutritional Sciences, University of Nottingham, Sutton Bonington Campus, Loughborough, Leicestershire LE125RD, UK

## Abstract

Expression of the brown adipocyte-specific gene, uncoupling protein 1 (UCP1), is increased by both PPAR*γ* stimulation and cAMP activation through their ability to stimulate the expression of the PPAR coactivator PGC1*α*. In HIB1B brown preadipocytes, combination of the PPAR*γ* agonist, rosiglitazone, and the cAMP stimulator forskolin synergistically increased UCP1 mRNA expression, but PGC1*α* expression was only increased additively by the two drugs. The PPAR*γ* antagonist, GW9662, and the PKA inhibitor, H89, both inhibited UCP1 expression stimulated by rosiglitazone and forskolin but PGC1*α* expression was not altered to the same extent. Reporter studies demonstrated that combined rosiglitazone and forskolin synergistically activated transcription from a full length 3.1 kbp UCP1 luciferase promoter construct, but the response was only additive and much reduced when a minimal 260 bp proximal UCP1 promoter was examined. Rosiglitazone and forskolin in combination were able to synergistically stimulate promoters comprising of tandem repeats of either PPREs or CREs. We conclude that rosiglitazone and forskolin act together to synergistically activate the UCP1 promoter directly rather than by increasing PGC1*α* expression and by a mechanism involving cross-talk between the signalling systems regulating the CRE and PPRE on the promoters.

## 1. Introduction

Nonshivering thermogenesis in brown adipose tissue (BAT) in response to a cold environment is initiated by sympathetic neural stimulation of *β*-adrenergic receptors on brown adipocytes which elevate intracellular cyclic AMP (cAMP) and, via the protein kinase A (PKA) pathway, increase the expression and thermogenic activity of uncoupling protein 1 (UCP1) [1]. UCP1 is BAT specific and responsible for uncoupling oxidative phosphorylation by enabling protons to return to the mitochondrial matrix without ATP synthesis, thereby producing heat. UCP1 expressing BAT has recently been identified in humans and has been proposed as a target for activation to increase energy expenditure and prevent or treat obesity [2]. 

UCP1 expression has been suggested to be regulated by the cAMP-inducible peroxisome proliferator activated coactivator 1*α* (PGC1*α*) which interacts with a BAT determination factor, PRDM16, to increase the expression of a number of BAT-selective genes including Cidea [3]. We have also shown that the cAMP-inducible transcription factor C/EBP*β* stimulates PGC1*α* expression in white and brown adipocytes by binding to the cAMP response element (CRE) on the PGC1*α* proximal promoter [4, 5] while others have demonstrated that PKA activation of PGC1*α* expression involves phosphorylation of p38 MAPK [6]. The PPAR*γ* ligand, rosiglitazone increases expression of PGC1*α* [7, 8] acting on a distal PPRE which binds PPAR*γ*/retinoid X receptor heterodimers and further positively autoregulates its own expression by coactivating PPAR*γ* responsiveness to rosiglitazone [7]. Furthermore, C/EBP*β* has been suggested to bind to PRDM16 to activate PGC1*α* expression during brown adipogenesis [9].

Therefore, UCP1 expression is thought to be regulated indirectly through an increased expression of PGC1*α* which then coactivates PPAR*γ* transactivation of the PPRE on the UCP1 enhancer [6]. cAMP response elements (CREs) have also been identified in the proximal promoter and a distal enhancer of UCP1 [6, 10], but the relative roles of direct and indirect interactions with the UCP1 promoter are uncertain. Furthermore, few studies have examined the interaction between cAMP and PPAR*γ* ligands. Here, we report that stimulation of the PKA and PPAR*γ* signaling pathways synergistically and directly stimulates transcription from the UCP1 promoter, due to the cross-talk between the two pathways.

## 2. Methods

### 2.1. Plasmids

The firefly luciferase reporter gene constructs containing the 3.1 kbp or 260 bp upstream of mouse UCP1 transcription site were kind gifts from Leslie P. Kozak, Pennington Biomedical Research Center, Louisana [10]. The 2.6 kbp PGC1*α*-pGL3-Luc containing 2600 bp insert size between +78 and −2533 with respect to mouse transcript start site was purchased from Addgene (UK), and the 264 bp (264 PGC1*α*-pGL3) from the region upstream of the rodent Pgc-1*α* transcription start site ligated to the pGL3-Basic vector (Promega) has been described [4]. The CRE positive vector (4 × CRE-Luc) that contains four repeat copies of the consensus CRE sequence upstream of a TATA box to drive expression of the firefly luciferase gene was purchased from Stratagene. The PPRE positive vector consisting of mouse PPRE × 3-TK-luc containing 3 direct repeat (DR1) of response elements (AGGACAAAGGTCA) upstream of a luciferase gene was purchased from Addgene (UK). The −2253-CRE-mut-PGC1*α*-Luc promoter construct was kindly given by F. Villarroya (University of Barcelona, Barcelona, Spain). The −2253-CRE-mut-PGC1*α*-Luc contains a point mutation at −146/−129 which was obtained by digestion of −2553-PGC1*α*-Luc with PvuII and ZraI and further ligation [7].

### 2.2. Cell Culture, Transfections, and Luciferase Assays

HIB-1B cells (kindly provided by B. Spiegelman) were maintained in DMEM with 10% FBS (Invitrogen) in 5% CO_2_. For transfections, HIB-1B cells were cultured to 80% confluence and then were transfected with pGL3 luciferase plasmids using Fugene 6 (Roche) according to the manufacturer's instructions. The pRL-SV40 construct (Promega) that carries renilla luciferase gene was cotransfected as an internal control for monitoring the transfection efficiency. Twenty-four hours later, cells were treated with DMSO (control), rosiglitazone (10 *μ*M) for 24 hours, or forskolin (10 *μ*M) for the final 12 hours of rosiglitazone treatment, in serum-free medium, before luciferase activity was measured using the Dual-Luciferase assay kit (Promega), as recommended by the manufacturer. Values were normalised relative to the renilla signal to allow for differences in transfection efficiency.

For mRNA expression studies, HIB-1B cells were grown to confluence and then treated with H89 (10 *μ*M) for 1 hour or GW9662 (30 *μ*M) for 3 hours prior to and during addition of rosiglitazone (Rosi) (10 *μ*M) for 24 hours or forskolin (Fosk) (10 *μ*M) for the final 3 hours of rosiglitazone treatment, before RNA extraction, as indicated. All drugs were added in serum-free medium. Controls were treated with DMSO.

### 2.3. Real-Time PCR

 Total RNA was extracted from cultured cells using TRI reagent (Sigma). Prior to RT-PCR, samples were treated with RNase-free DNase to remove contaminating genomic or plasmid DNA. cDNA was generated using the cDNA synthesis kit from Qiagen. Quantitative real-time PCR (qRT-PCR) was performed using Sybr green according to the manufacturer's instructions (Roche). The sequences of the primers used for real-time PCR are given in Table 1. Expression levels for all genes were normalized to expression of the house keeping gene, 36B4.

### 2.4. Statistical Analysis

To examine the effects of agonists (forskolin, rosiglitazone) and antagonists (GW9662, H89) as well as interaction effects (agonists × antagonists) on mRNA levels or luciferase reporter activities of all groups, a two-way ANOVA (SPSS, v17) was performed. The comparisons between agonist and agonist + antagonist treated cells or between wildtype 2.6 kb PGC1*α*-Luc and CRE-mut-PGC1*α*-Luc transfected cells were determined by *t* test.

## 3. Results 

### 3.1. Synergistic Increase in UCP1, but Not PGC1*α*, Cidea, or PRDM16 in Response to Combined Forskolin and Rosiglitazone Is Inhibited by a PKA Inhibitor (H89) and a PPAR*γ* Antagonist (GW9662)

**Figure 1:**
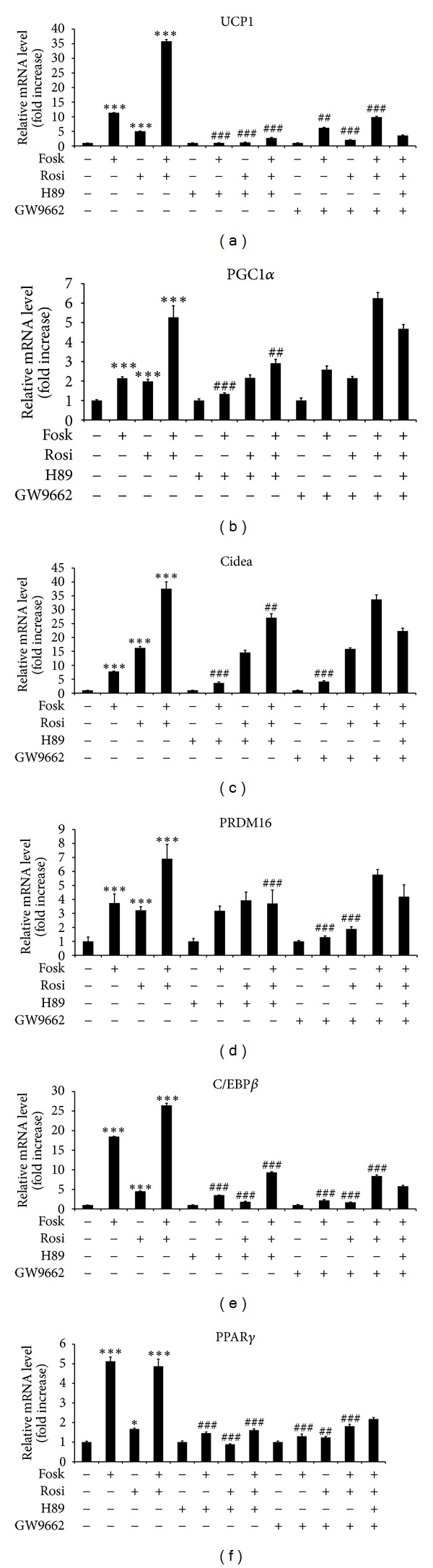
The effect of forskolin and rosiglitazone on mRNA expression of (a) UCP1, (b) PGC1*α*, (c) Cidea, (d) PRDM16, (e) C/EBP*β*, and (f) PPAR*γ* is mediated by PKA and PPAR*γ* dependent pathways. HIB-1B cells were grown to confluence and then treated with H89 (10 *μ*M) for 1 hour or GW9662 (30 *μ*M) for 3 hours prior to and during addition of rosiglitazone (Rosi) (10 *μ*M) for 24 hours, or forskolin (Fosk) (10 *μ*M) for the final 3 hours of rosiglitazone treatment, before RNA extraction, as indicated. All drugs were added in serum-free medium. Controls were treated with DMSO. Gene expression levels were analysed by quantitative real-time PCR and normalized against 36B4 expression. Error bar means the mean ± SEM of triplicate observations within a single experiment performed in triplicate. *** Significant difference *P* < 0.001 with respect to control; ^###^ significant difference *P* < 0.001 due to H89 or GW9662 for each experiment.

Addition of forskolin for 3 h and rosiglitazone for 24 h increased UCP1 mRNA expression by 12-fold (*P* < 0.001) and 5.5-fold (*P* < 0.001), respectively, but when forskolin was added during the last 3 h of incubation with rosiglitazone, a synergistic 40-fold increase (*P* < 0.001) was observed, relative to control confluent HIB1B cells (Figure 1(a)). Addition of the PKA inhibitor H89 significantly increased by 4-fold (*P* < 0.001) the basal expression of UCP1 mRNA but completely blocked the stimulatory effect of forskolin (*P* < 0.001) and inhibited the synergistic response to forskolin plus rosiglitazone by 75% (*P* < 0.001). H89 also suppressed the UCP1 mRNA response to rosiglitazone by 75% (*P* < 0.001). The PPAR*γ* antagonist GW9662 doubled the basal levels of UCP1 mRNA but inhibited the response to forskolin and rosiglitazone by 50–75% (*P* < 0.001). A mixture of GW9662 and H89 decreased UCP1 mRNA by 88% in response to forskolin plus rosiglitazone, relative to control cells (*P* < 0.001).

In contrast, PGC1*α* mRNA was upregulated two-fold by forskolin or rosiglitazone treatment (*P* < 0.001; Figure 1(b)) and only additively by combined forskolin and rosiglitazone treatment. Addition of H89 downregulated the PGC1*α* expression response to forskolin by 77% (*P* < 0.001), but surprisingly, addition of GW9662 did not alter PGC1*α* expression in the presence of forskolin, or rosiglitazone, separately or in combination. These results suggested that the action of cAMP and PPAR*γ* stimulation on the initiation of UCP1 expression was directly targeting UCP1 rather than indirectly acting on PGC1*α* expression.

We next examined whether the brown selective genes Cidea and PRDM16 were responsive to PKA and PPAR*γ* modulation. Similar to UCP1, expression of Cidea was increased by both forskolin (*P* < 0.001) and rosiglitazone (*P* < 0.001) by 8-fold and 18-fold, respectively, and when forskolin and rosiglitazone were combined together, there was a synergistic 40-fold stimulatory effect (*P* < 0.001) relative to control cells (Figure 1(c)). In contrast to UCP1, although H89 caused a significant 65% reduction (*P* < 0.001) in Cidea expression in response to forskolin, there was no effect of H89 on Cidea expression in response to rosiglitazone and only a small 25% inhibition of the response to combined forskolin plus rosiglitazone (*P* < 0.01) was observed. Furthermore, GW9662 did not alter Cidea expression in response to rosiglitazone or forskolin plus rosiglitazone although forskolin stimulated Cidea expression was suppressed by 21% (*P* < 0.01). Again in contrast to UCP1, PRDM16 mRNA levels were induced by only 3-fold (*P* < 0.001) in response to forskolin or rosiglitazone and only additively in response to forskolin plus rosiglitazone (*P* < 0.001) (Figure 1(d)), relative to control cells. Addition of H89 did not alter the response to either forskolin or rosiglitazone, but the response to combined forskolin and rosiglitazone was inhibited by 58% (*P* < 0.05). Addition of the PPAR*γ* antagonist, GW9662, inhibited PRDM16 expression in response to either forskolin or rosiglitazone by 56–60% (*P* < 0.001), but GW9662 failed to inhibit the PRDM16 response to forskolin and rosiglitazone in combination. Expression of the adipogenic differentiation marker gene aP2, in response to stimulation by forskolin and rosiglitazone, was similarly less sensitive to H89 and GW9663, compared to UCP1 (results not shown). These results again suggest that the action of cAMP and PPAR*γ* stimulation on the initiation of UCP1 expression was directly targeting the UCP1 promoter.

### 3.2. Effect of a PKA Inhibitor (H89) and a PPAR*γ* Antagonist (GW9662) on C/EBP*β* and PPAR*γ* Expression in Response to Forskolin and Rosiglitazone Is Different to Responses Observed on UCP1

Our attention was then drawn to the effect of rosiglitazone and forskolin on the expression of C/EBP*β* and PPAR*γ*, genes which have been reported to regulate UCP1 expression. C/EBP*β* mRNA was increased by 18-fold (*P* < 0.001) in response to forskolin and 4.5-fold (*P* < 0.001) in response to rosiglitazone, relative to control cells (Figure 1(e)). In addition, a 26-fold additive response to combined forskolin and rosiglitazone (*P* < 0.01) in C/EBP*β* mRNA was observed. H89 blocked forskolin stimulated C/EBP*β* expression by 82% (*P* < 0.001), rosiglitazone induced expression by 32% (*P* < 0.01), and the combined effect of forskolin plus rosiglitazone was inhibited by 68% (*P* < 0.001). GW9662 blocked forskolin stimulated C/EBP*β* expression by 93% (*P* < 0.001), rosiglitazone induced C/EBP*β* expression by 49% (*P* < 0.01), and inhibited the additive activation by forskolin and rosiglitazone combination on C/EBP*β* expression by 50% (*P* < 0.001).

PPAR*γ* mRNA was increased by 5.2-fold (*P* < 0.001) in response to PKA activation with forskolin and 1.7-fold (*P* < 0.05) by rosiglitazone, but there was no further response when forskolin and rosiglitazone were combined (Figure 1(f)). Addition of H89 significantly decreased the response to forskolin by 70% (*P* < 0.001). GW9662 reduced PPAR*γ* expression by 90% (*P* < 0.001) in response to forskolin and by 73% (*P* < 0.001) and 70% (*P* < 0.001), respectively, in response to rosiglitazone and forskolin plus rosiglitazone, respectively.

Therefore, the expression of PPAR*γ* and C/EBP*β* was predominantly increased by forskolin with only a small effect of combining forskolin and rosiglitazone, but these effects could be suppressed by both PKA inhibition (H89) and the PPAR*γ* antagonist GW9662. Combined the results suggest that the effects of the inhibitors and stimulators on UCP1 expression could not be completely explained by changes in the expression of regulatory genes measured. Similar conclusions were drawn when expression levels of CtBP1, CtBP2, and RIP140 were measured (result not shown).

### 3.3. Synergistic Activation of UCP1 and PGC1*α* Transcription by Combined Forskolin and Rosiglitazone Requires the Full Length Promoters

**Figure 2:**
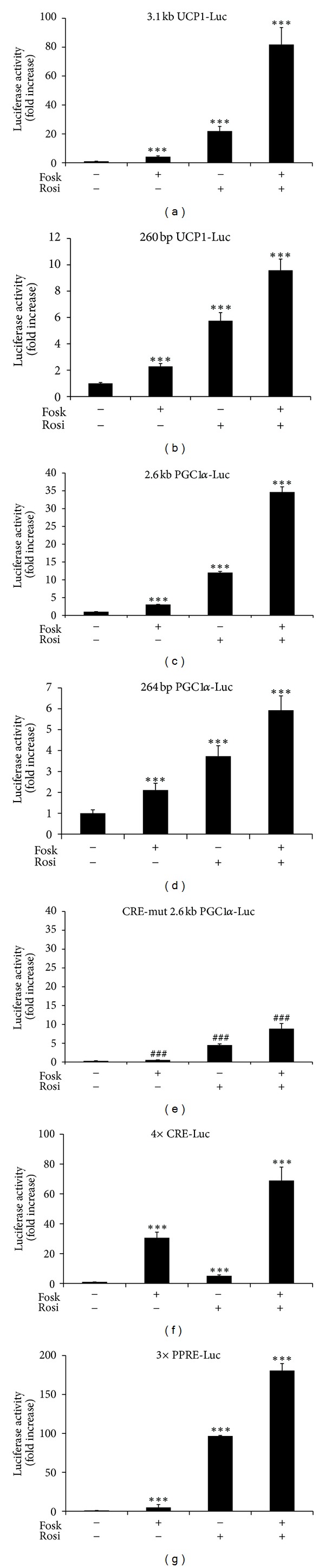
Luciferase reporter assay showing the effect of forskolin and rosiglitazone on (a) 3.1 kbp UCP1-Luc, (b) 260 bp UCP1-Luc, (c) 2.6 kbp PGC1*α*-Luc, (d) 264 bp PGC1*α*-Luc, (e) CRE-mut 2.6 kbp PGC1*α*-Luc, (f) 4 × CRE-Luc, and (g) 3 × PPRE-Luc, promoter activities. HIB-1B cells were grown to confluence and then transiently transfected with the above -pGL3-Luc reporter construct, as indicated. Twenty-four hours later, cells were treated with DMSO (control), rosiglitazone (10 *μ*M) for 24 hours, or forskolin (10 *μ*M) for the final 12 hours of rosiglitazone treatment, in serum-free medium, before luciferase activity was measured. Values were expressed as fold increase of ratio of firefly to renilla. Error bar means the mean ± sem. of three observations within a single experiment performed in triplicate. *** Significant difference *P* < 0.001, respectively, with respect to control; ^###^ significant difference *P* < 0.001 from wildtype due to the mutation of CRE.

We next examined the effect of PKA and PPAR*γ* activation on full length and truncated UCP1 and PGC1*α* promoter luciferase reporter constructs transfected into confluent HIB1B cells. Addition of rosiglitazone for 24 h or forskolin for 12 h significantly induced transcriptional activity of the full length 3.1 kb UCP1 promoter reporter construct by 22-fold and 4.1-fold (*P* < 0.001), respectively, and when the drugs were combined, synergistically increased transcription by 82-fold (Figure 2(a); *P* < 0.001). By comparison, transcription from the proximal 260 bp UCP1 promoter was only stimulated 2-fold and 6-fold, by forskolin and rosiglitazone, respectively (*P* < 0.001), and combined addition of the two drugs increased reporter activity by 10-fold (*P* < 0.001; Figure 2(b)). The 260 bp promoter contains only one CRE, no PPRE, but is activated by forskolin to approximately the same extent, as the 3.1 kb UCP1 promoter reporter construct [11].

We next examined a PGC1*α*-Luc promoter reporter. Forskolin addition significantly doubled (*P* < 0.001) transcription from the full length 2.6 kb PGC1*α* promoter while addition of rosiglitazone stimulated by 12-fold (*P* < 0.001; Figure 2(c)). When forskolin and rosiglitazone were combined together, there was a synergistic 35-fold increase in the 2.6 kb PGC1*α* transcription activity (*P* < 0.001). As observed with the proximal UCP1 promoter, there were significant (*P* < 0.001) effects of forskolin and rosiglitazone, alone or in combination, on transcription from the proximal 264 bp PGC1*α*-Luc minimal promoter, even though this contains only a CRE and no PPRE [4], but transcription activities and fold responses were 3–6-fold less compared with the full length 2.6 kb PGC1*α*-Luc (Figure 2(d)). Mutation of the CRE on the 2.6 kb PGC1*α* promoter significantly decreased both basal expression and the response to forskolin and rosiglitazone, alone or in combination, by between 63% and 80% (*P* < 0.001), compared to wildtype 2.6 kb PGC1*α* (Figure 1(g)). 

When reporter constructs under the control of promoters containing repeats of either CRE (4 × CRE-Luc) or PPRE (PPRE × 3-Luc) were examined, as expected, forskolin stimulated mainly the CRE-Luc and rosiglitazone stimulated the PPRE-Luc (*P* < 0.001; Figures 2(e) and 2(f)). However, there was significant stimulation of the PPRE-Luc by forskolin (*P* < 0.001), of the CRE-Luc by rosiglitazone (*P* < 0.001), and a synergistic response to combined forskolin plus rosiglitazone for both the CRE-Luc and PPRE-Luc (*P* < 0.001; Figures 2(e) and 2(f)). These results clearly suggest that the synergistic response in the UCP1 promoter activity to combined forskolin and rosiglitazone involves interaction between pathways targeting the CREs and PPREs on the UCP1 promoter. 

## 4. Discussion

Expression of UCP1 in brown adipocytes can be induced *in vitro* and *in vivo* by drugs that activate PKA and PPAR*γ*, but few studies have examined the interaction between these pathways. This study compares the expression of a number of brown adipogenic genes in response to PKA and PPAR*γ* stimulators and inhibitors in brown HIB-1B preadipocytes and demonstrates that the synergistic induction of UCP1 expression by combined PKA activation and PPAR*γ* agonist treatment involves cross-talk between these pathways. This conclusion is based on the abilities of a PKA inhibitor to suppress responses to the PPAR*γ* agonist and inhibition of PKA stimulation by a PPAR*γ* antagonist. The responses of other brown adipogenic genes (Cidea, aP2) and regulators of brown adipogenesis (PGC1*α*, PRDM16, PPAR*γ* C/EBP*β*) to PKA or PPAR*γ* activation/inhibition were different from UCP1, and, combined with reporter studies, we provide evidence that these responses to PKA activation and the PPAR*γ* agonist treatment are targeted at the UCP1 promoter. 

Combined addition of forskolin plus rosiglitazone resulted in a robust synergistic induction of UCP1 mRNA expression in undifferentiated confluent HIB-1B cells. A previous study was able to demonstrate a synergistic response in UCP1 and PGC1*α* expression to acute (2 hours) noradrenaline and chronic (continuously in the culture medium) rosiglitazone in differentiated mouse primary brown adipocytes [12]. Our luciferase reporter studies demonstrated that this synergistic response required the full length 3.1 kbp UCP1 promoter containing both the enhancer and proximal promoter elements since a reporter construct containing only the proximal 260 bp promoter was nearly 10-fold less responsive, and stimulation by forskolin + rosiglitazone was only additive. The stimulatory effect of rosiglitazone on the proximal UCP1 promoter was greater than forskolin despite this construct containing only a CRE and no PPRE. The ability of rosiglitazone to stimulate transcription at CREs was confirmed using an artificial promoter reporter containing tandem CRE repeats, as well as a 264 bp PGC1*α* proximal promoter reporter, neither of which contain a PPRE. Furthermore, when the CRE on the PGC1*α* 2.6 kbp promoter was mutated, there was a marked diminution of the responses both to rosiglitazone alone and combined forskolin + rosiglitazone. A previous study [13] using a reporter construct containing the 220 bp enhancer linked to a 73 bp minimal UCP1 promoter reporter construct similarly observed synergistic activation by combined PPAR*γ* agonist and dibutyryl cAMP, but no responses were observed with the 73 bp minimal promoter which does not contain a CRE. Furthermore, the increase in UCP1 mRNA expression by rosiglitazone treatment was abolished by the PKA inhibitor, H89, and this effect was greatest when forskolin was added in combination. This inhibitory effect of H89 on gene expression in response to rosiglitazone appeared to be greatest for UCP1 and was not observed to the same extent with other genes involved in brown adipogenesis (Cidea, PRDM16, C/EBP*β*, and PGC1*α*). The evidence therefore suggests that rosiglitazone-stimulated UCP1 expression, which is synergistically increased by forskolin, is at least partly due to increased activation of the CREs. 

The mechanism by which rosiglitazone is able to stimulate the PKA pathway is not known. Rosiglitazone is a PPAR*γ* ligand which increases the interaction of PPAR*γ* with coactivators such as steroid receptor coactivator-1 (SRC-1) and p300/CBP (CREB binding protein) containing histone acetyl transferase activity, which is important for remodelling chromatin to increase the transcriptional activity of nuclear receptors [14]. GW9662 blocks the recruitment of these coactivators [14], and therefore the stimulatory effect of rosiglitazone on transactivation at the CRE may be caused by chromatin remodelling. In the present study, rosiglitazone strongly stimulated an artificial reporter containing tandem PPREs and in the study of [13], mutation of the PPRE on the UCP1 enhancer abolished responses to both PKA activation and a PPAR*γ* agonist. However, there has been a report in primary brown adipocytes that rosiglitazone stimulates p38 MAPK phosphorylation in a non-PPAR*γ* manner [8] which would explain why the expression of PGC1*α* appeared to be induced by rosiglitazone even in the presence of GW9662 (Figure 1(b)). PKA-dependent stimulation of PGC1*α* expression has been shown to involve p38-MAPK-dependent phosphorylation of ATF2 [6, 10]. Therefore, rosiglitazone activation of p38 MAPK would be likely to increase ATF2 phosphorylation and stimulate transcription from the CREs in the PGC1*α* promoter, as suggested for the effects of PKA activation.

The ability of rosiglitazone to stimulate the PPRE-Luc reporter activity was markedly stimulated by addition of forskolin, suggesting that increasing PKA activity was able to synergise with rosiglitazone by increasing transactivation of the PPRE on the UCP1 and PGC1*α* promoters. This proposal was supported by evidence that forskolin stimulation of UCP1 mRNA expression was partly decreased by addition of the PPAR*γ* antagonist GW9662. Recently, it has been demonstrated that *β*-adrenergic agonist stimulation of UCP1 expression in HIB-1B cells is associated with an increase in PGC1*α* and PPAR*γ* binding to the PPRE on the UCP1 enhancer [15]. PKA induced binding of C/EBP*β* to the PPAR*γ*2 promoter during adipogenesis is known to increase chromatin accessibility of transcription factors to the promoter [16], emphasising the importance of chromatin remodelling in transcriptional responses to PKA stimulation. Further studies are required to establish functional crosstalk between the pathways targeting the CREs and PPREs on the UCP1 promoter, for example, knocking down PPAR*γ* or C/EBP*β* expression to establish if this blocks induction by forskolin or rosiglitazone, respectively.

In the present study, forskolin stimulated PGC1*α* expression by activating PKA as indicated by the complete suppression with H89, and the stimulatory effect of forskolin was almost totally blocked when the CRE in the proximal promoter reporter construct was mutated, in agreement with previous reports [6, 7]. Forskolin also induced the expression of PPAR*γ* and C/EBP*β*, in a PKA-dependent manner which is consistent with their role as transcription factors stimulating both UCP1 and PGC1*α* expression [4, 6, 7, 9]. C/EBP*β*, which is enriched in BAT compared to WAT, is sensitive to cold stimulation in BAT and forskolin treatment in HIB-1B cells, has been proposed to play a key role in the cAMP induction of UCP1 and PGC1*α* expression in a white adipocyte cell line [4, 9]. Therefore, our data showing that C/EBP*β* expression is cAMP dependent and blunted by H89 is in accordance with previous studies. C/EBP*β* and PRDM16 have been shown to form a transcriptional complex which increases PGC1*α* and UCP1 expression [9]. However, the pattern of expression responses of PGC1*α*, C/EBP*β*, PPAR*γ*, and PRDM16 to combined PKA stimulation and PPAR*γ* agonist/antagonist treatment was generally different from responses in UCP1 expression, suggesting that changes in the expression of these regulators were not major factors in the control of UCP1 transcription. This notion was supported by our time course studies demonstrating that forskolin stimulated UCP1 expression in confluent undifferentiated HIB-1B cells within 24 hours while changes in PGC1*α*, C/EBP*β*, and PRDM16 expression were either delayed for 72 hours or absent (results not shown). The control of UCP1 expression has previously been dissociated from that of PGC1*α* in *β*1/*β*2/*β*3-adrenoceptor knockout (*β*-less) brown adipocytes in primary culture [17]. Furthermore, in PGC1*α* depleted cells, the amounts of several brown fat-selective genes are not affected [18] supporting a role for PGC1*α* as a coactivator of PPAR*γ* in response to cold but not for brown adipose differentiation.

The synergistic effects of combined PPAR*γ* agonist and PKA stimulation on UCP1 expression appear to be physiologically relevant as surgical denervation of BAT pads in rats prevented maximal brown adipogenic gene expression in response to chronic rosiglitazone treatment [19]. Furthermore, PPAR*γ* ligand treatment can increase UCP1 and PGC1*α* expression in rodent white adipose depots [20] or preadipocytes from WAT in mice [21] and humans [22].

## 5. Conclusions

The synergistic stimulation of UCP1 expression by combined forskolin and rosiglitazone appears to be directly targeting the UCP1 promoter and involves cross-talk between their PKA and PPAR*γ* signalling systems. This study suggests that treating obesity by increasing energy expenditure as a result of brown adipose thermogenesis is more likely to be successful by combined drug therapy aimed at stimulating both PKA and PPAR*γ* signalling pathways.

## Figures and Tables

**Table 1:** The sequences of primers for the real-time PCR.

Gene	Forward primer	Reverse primer
PGC1*α*	TGAGAGACCGCTTTGAAGTTTTT	CAGGTGTAACGGTAGGTGATGAAA
PRDM16	TCTTACTTCTCCGAGATCCGAAA	GATCTCAGGCCGTTTGTCCAT
C/EBP*β*	AGCGGCTGCAGAAGAAGGT	GGCAGCTGCTTGAACAAGTTC
UCP1	GCCATCTGCATGGGATCAA	GGTCGTCCCTTTCCAAAGTG
PPAR*γ*	GTGCCAGTTTCG ATCCGT AGA	GGCCAGCATCGTGTAGATA
aP2	AACACCGAGATTTCC	ACACATTCCACCACCAG
Cidea	ACAGAAATGGACACCGGGTAGT	CGAAGGTGACTCTGGCTATTCC
36B4	TCCAGGCTTTGGGCATCA	TTATCAGCTGCACATCACTCAGAAT

## Abbreviations

CRE: Cyclic adenosine monophosphate response element
BAT: Brown adipose tissue
WAT: White adipose tissue
UCP-1: Uncoupling protein-1
PPRE: Peroxisome proliferator activated receptor response element
PGC-1: Peroxisome proliferator activated receptor gamma coactivator 1.
